# Glucose transporter GLUT1 expression and clinical outcome in solid tumors: a systematic review and meta-analysis

**DOI:** 10.18632/oncotarget.15171

**Published:** 2017-02-07

**Authors:** Ji Wang, Chenyang Ye, Cong Chen, Hanchu Xiong, Binbin Xie, Jichun Zhou, Yongxia Chen, Shu Zheng, Linbo Wang

**Affiliations:** ^1^ Department of Surgical Oncology, Sir Run Run Shaw Hospital, College of Medicine, Zhejiang University, Hangzhou, Zhejiang, 310016, China; ^2^ Biomedical Research Center and Key Laboratory of Biotherapy of Zhejiang Province, Hangzhou, Zhejiang, 310016, China; ^3^ Cancer Institute (Key Laboratory of Cancer Prevention and Intervention, China National Ministry of Education), 2nd Affiliated Hospital, School of Medicine, Zhejiang University, Hangzhou, Zhejiang, 310009, China; ^4^ Reseach Center for Air Pollution and Health, School of Medicine, Zhejiang University, Hangzhou, Zhejiang, 310009, China

**Keywords:** GLUT1, solid tumors, prognosis, overall survival, disease-free survival

## Abstract

Glucose transporter 1 (GLUT1), the uniporter protein encoded by the *SLC2A1* gene, is a key rate-limiting factor in the transport of glucose in cancer cells, and frequently expressed in a significant proportion of human cancers. Numerous studies have reported paradoxical evidence of the relationship between GLUT1 expression and prognosis in solid human tumors. To address this discrepancy, we conducted a thorough search of Pubmed and Web of Science for studies evaluating the expression of GLUT1 and overall survival (OS) and disease-free survival (DFS) in patients with solid cancer from 1993 to April 2016. Data from published researches were extracted and computed into odds ratio (OR). A total of 26 studies including 2948 patients met our search criteria and were evaluated. Overexpression of GLUT1 was found to significantly correlate with poor 3-year OS (OR: 2.86; 95% CI, 1.90–4.32, *P* < 0.00001) and 5-year OS (OR: 2.52; 95% CI, 1.75–3.61, *P* < 0.00001) of solid tumors. Similar results were observed when analysis of DFS was performed. Subgroup analysis revealed that elevated GLUT1 expression was associated with worse prognosis of oral squamous cell carcinoma and breast cancer. Taken together, overexpression of GLUT1 is correlated with poor survival in most solid tumors, suggesting that the expression status of GLUT1 is a vital prognostic indicator and promising therapeutic target in solid tumors.

## INTRODUCTION

Malignant cells are known to reprogram their metabolism to boost the rapid growth, proliferation and long-lasting maintenance [1, 2]. The common features of this increased metabolism are elevated glucose uptake and lactic fermentation of glucose even under aerobic condition, which is termed “the Warburg effect” [2, 3]. The increased glucose uptake in malignant tumors is largely dependent on specific transmembranous glucose transporter proteins (GLUTs). Glucose transporter 1 (GLUT1), also named facilitates glucose transporter member 1 or solute carrier family 2 (SLC2A1), is a uniporter protein in humans encoded by the *SLC2A1* gene [4]. In normal tissues, GLUT1 is limited to be expressed on erythrocytes and endothelial cells in the blood-brain barriers [5]. Recently, GLUT1 has been demonstrated to be a pivotal rate-limiting element in the transport of glucose in malignancy cells and overexpressed in different types of human cancers [6–10]. A plenty of researches showed that GLUT1 is involved in the progression and metastasis of cancer cell [11, 12] In addition, overexpression of GLUT1 is correlated with vascular invasion, microvessel density and depth of invasion in carcinomas [13]. In light of the promoting role of GLUT1 in tumor metabolism and development, targeting GLUT1 for therapeutics and prevention might be conducive.

The correlation between GLUT1 expression and prognosis in cancer patients has been investigated. A myriad of studies showed that elevated expression level of GLUT1 in malignant tumors was correlated with poor clinical outcomes in patients with diverse types of solid tumors such as lung cancer [14, 15], breast cancer [16, 17], esophageal cancer [18], hepatocellular carcinoma [10], gallbladder carcinoma [19], colorectal cancer [20–23], oral squamous cell carcinoma [24–28], bladder cancer [29], ovarian cancer [30], head and neck squamous cell carcinoma [31], and salivary gland tumor [32]. However, some other researches showed overexpression of GLUT1 was related to favorable clinical outcome [33]. In addition, several researches revealed that the expression of GLUT1 was not significantly associated with prognosis of patients [19, 34–37]. Taken together, the exact clinical and prognostic merit of GLUT1 overexpression in various solid tumors remains unclear.

We herein performed an exhaustive meta-analysis to appraise the prognostic significance of GLUT1 overexpression in solid tumors. The objective of our analysis was to value the relationship of elevated GLUT1 expression status with prognostic outcomes in solid human tumors, and illustrate the clinical value of GLUT1 as a prognostic indicator and potential therapeutic target for malignant tumor patients.

## RESULTS

### Search results and study characteristics

**Figure 1:**
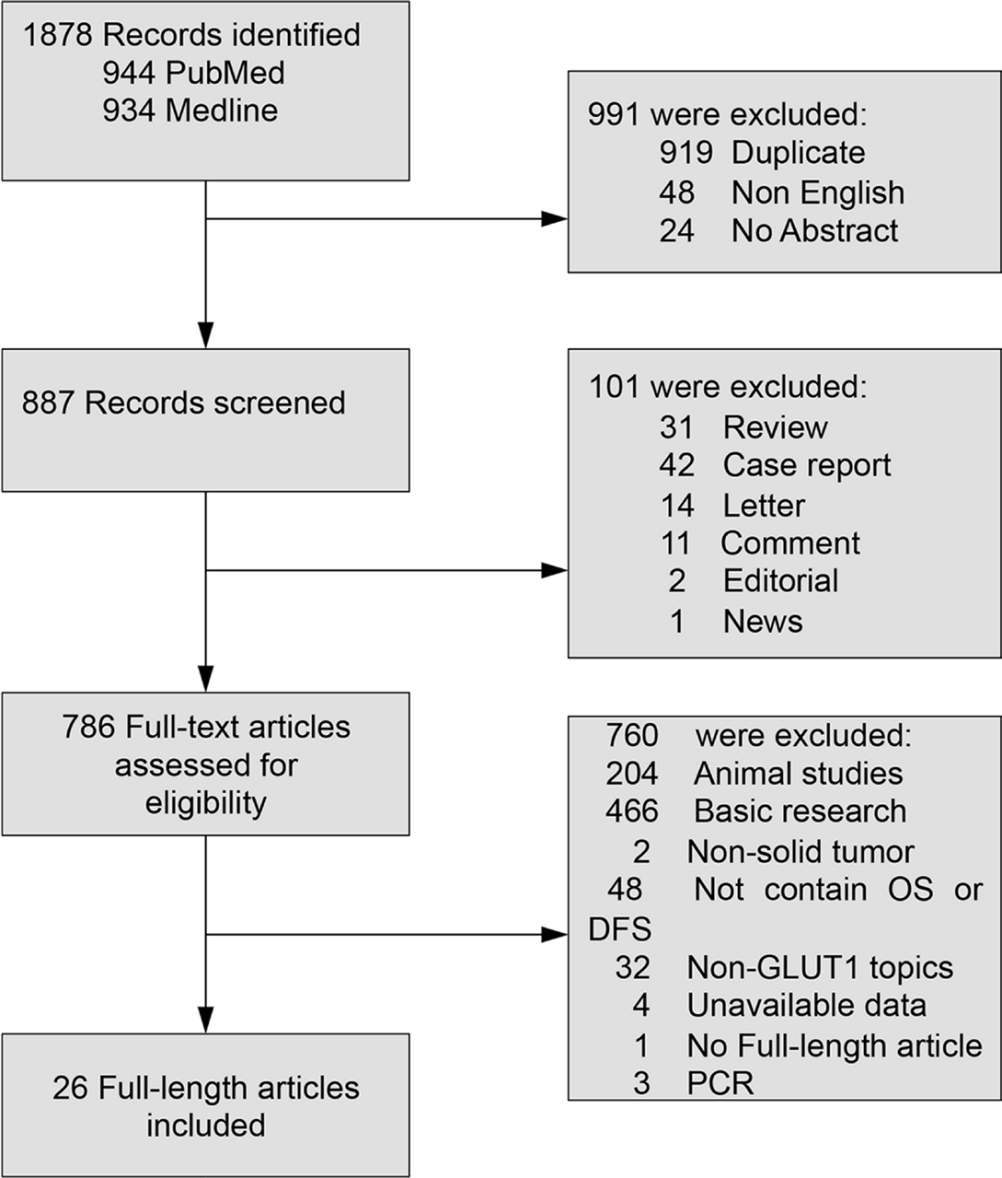
Flowchart of study selection in the meta-analysis OS: overall survival; DFS: disease-free survival.

26 researches with a total of 2948 patients were ultimately involved (Figure 1). The main characteristics of included researches were presented in Table 1. Five researches appraised colorectal cancer [20–23, 33], five evaluated orals squamous cell carcinoma [24–28], three evaluated cervical cancer [35, 36, 38], two studies evaluated lung cancer [14, 15], two evaluated breast cancer [16, 17], two studies evaluated pancreatic cancer [19, 34] and one each evaluated esophageal cancer [18], hepatocellular carcinoma[10], gallbladder carcinoma [19], bladder cancer [29], ovarian cancer [30], head and neck squamous cell carcinoma [31], renal cancer [37], and salivary gland tumor [32]. All these 26 studies evaluated GLUT1. As for the region, 12 studies were conducted in Asia, seven studies in America, and seven studies in Europe.

**Table 1:** Characteristics of studies included in the meta-analysis

References	Country	Type of cancer	Patient No.	Age,median (range)	Male/ Female	Stage	Follow up, months (Range)	Patients setting
Studies including OS								
Basturk, O., et al. (2011)	USA	PC	94	NR	NR/NR	I–IV	NR	surgical
Chen, B., et al. (2015)	China	BC	122	NR	0/122	I–IV	NR	surgical
Cho, H., et al. (2013)	Korea	EOC	50	NR	0/50	FIGO stage I–IV	31.60 (26.77–36.43)	surgical
Cleven, A. H. G., et al. (2007)	Netherlands	CRC	133	NR	55/78	I-IV	NR	surgical
Cooper, R., et al. (2003).	Turkey	CRC	43	57 (21–80)	29/14	Dukes stage (B-C)	46 (8–102)	surgical
Eckert, A. W., et al. (2008).	Germany	OSCC	42	61 (26–83)	33/9	I–IV	NR	surgical
Furudoi, A., et al. (2001)	Japan	CRC	152	63.3	94/58	Dukes stage (B-D)	63.2	surgical
Goos, J. A. C. M., et al. (2016)	Netherlands	CRC	350	NR	NR/NR	NR	NR	surgical
Haber, R. S., et al. (1998)	USA	CRC	112	67 ± 10	60/52	Dukes stage (A-D)	84 (3–152)	surgical
Kaira, K., et al. (2013)	Japan	LC	34	70 (51–78)	24/10	I–II	24 (6–87)	surgical
Kang, S. S., et al. (2002)	Korea	BC	100	48.3 (23–74)	0/100	NR	57.4 (49–67)	surgical
Kim, B. W., et al. (2013)	Korea	CC	179	43.8 (19-83)	0/179	FIGO stage I.II.IV	55.6 (6–60)	surgical
Kitamura, K., et al. (2011)	Japan	HC	63	65.6 (32–80)	48/15	I–IV	38 (2.5–66.7)	surgical
Kunkel, M., et al. (2003)-1 stage I–II	USA	OSCC	118	59 (35–80)	88/30	I–IV	38 (7–60)	surgical
Kunkel, M., et al. (2003)-2 stage III–IV	USA	OSCC	118	59 (35–80)	88/30	I–IV	38 (7–60)	surgical
Kunkel, M., et al. (2007)	USA	OSCC	40	52 (34–72)	7/33	I–IV	62 (25–106)	surgical
Lidgren, A., et al. (2008)-1cRCCs	Sweden	RCC	187	65 (25–87)	108/79	I–IV	42 (0–281)	surgical
Lidgren, A., et al. (2008)-2pRCCs	Sweden	RCC	187	65 (25–87)	108/79	I–IV	42 (0–281)	surgical
Mayer, A., et al. (2005)	Germany	CC	47	NR	0/47	FIGO IB-IVB	28 (4–95)	surgical& non-surgical
Mori, Y., et al. (2007)	Japan	SGC	87	51.6 (14–82)	36/51	NR	NR	surgical
Ohba, S., et al. (2010)	Japan	OSCC	24	61 (34–88)	14/10	NR	17.6 (3–37)	surgical
Osugi, J., et al. (2015)	Japan	LC	134	70 (48–87)	92/42	I–III	60.0 (1–60)	surgical
Sung, J.-Y., et al. (2010)-1	Korea	AVC	67	NR	34/33	I–IV	73 (1–264)	surgical
Sung, J.-Y., et al. (2010)-2	Korea	PC	52	NR	33/19	II–IV	28 (2–244)	surgical
Sung, J.-Y., et al. (2010)-3	Korea	EBDC	121	NR	84/37	I–III	45 (1–235)	surgical
Sung, J.-Y., et al. (2010)-4	Korea	GBC	115	NR	56/58	I–III	36 (1–160)	surgical
Tohma, T., et al. (2005)	Japan	ESCC	63	62.0 (40–78)	55/8	NR	36.4 (2–145)	surgical
Younes, M., et al. (2001)	USA	TCCB	40	65 (49–82)	NR/NR	NR	48 (5–60)	surgical
Studies including DFS								
Airley, R., et al. (2001)	UK	CC	121	NR	0/121	FIGO stage I–IV	NR	non-surgical
Baschnagel, A. M., et al. (2015)	USA	HNSCC	97	61 (42–85)	NR/NR	NR	35 (1–93)	non-surgical
Chen, B., et al. (2015)	China	BC	122	NR	0/122	I–IV	NR	surgical
Grimm, M., et al. (2014)	USA	OSCC	161	NR	122/39	Stage I–IV	52.26 (46.21–58.31)	surgical
Kang, S. S., et al. (2002)	Korea	BC	100	48.3 (23–74)	0/100	NR	57.4 (49–67)	surgical
Kim, B. W., et al. (2013)	Korea	CC	179	43.8 (19–83)	0/179	FIGO stage I.II.IV	55.6 (6–60)	surgical
Kitamura, K., et al. (2011)	Japan	HC	63	65.6 (32–80)	48/15	I–IV	38 (2.5–66.7)	surgical
Osugi, J., et al. (2015)	Japan	LC	134	70 (48–87)	92/42	I–III	60.0 (1–60)	surgical

### Assessment and expression status of GLUT1

A depiction of primary antibodies, and cut-off values of GLUT1 utilized in the eligible researches was presented in Table 2. Different antibodies were utilized for the appraisement of GLUT1 expression by immunohistochemistry (IHC). For anti-GLUT1 antibody, five researches utilized clone MYM, four researches utilized clone A3536, one research each used clone AB15309, SPM498, OH-217, and fourteen researches did not mention the antibody clone. Among the groups identified as GLUT1 positive, the median expression of GLUT1 in solid tumors was 50.00%, range from 17.87% to 84.96%.

**Table 2:** Evaluation of human GLUT1 by IHC in the selected studies

References	Type of tumor	Cutoff	Antibody (Clone)
Airley, R., et al. (2001)	CC	IHC score ≥ 1	anti-GLUT1(NR); Alpha Diagnostic International
Baschnagel, A. M., et al. (2015)	HNSCC	IHC score ≥ 3	anti-GLUT1(NR); Abcam
Basturk, O., et al. (2011)	PC	IHC score ≥ 1	anti-GLUT1(NR); polyclonal antibody; DAKO
Chen, B., et al. (2015)	BC	IHC score ≥ 2	NR
Cho, H., et al. (2013)	EOC	IHCscore > 3.85	anti-GLUT1(NR); monoclonal antibody; R&D Systems
Cleven, A. H. G., et al. (2007)	CRC	IHC >5%	anti-GLUT1(A3536); polyclonal antibody; DAKO
Cooper, R., et al. (2003).	CRC	IHC >1%	anti-GLUT1(NR); Alpha Diagnostic International
Eckert, A. W., et al. (2008).	OSCC	IHC score ≥ 9	anti-GLUT1(NR); Acris antibodies
Furudoi, A., et al. (2001)	CRC	IHC > 30%	anti-GLUT1(MYM); polyclonal antibody; DAKO
Goos, J. A. C. M., et al. (2016)	CRC	NR	anti-GLUT1(NR); polyclonal antibody; Abcam
Grimm, M., et al. (2014)	OSCC	IHC > 10%	anti-GLUT1(NR); polyclonal antibody; Dako
Haber, R. S., et al. (1998)	CRC	IHC > 50%	NR
Kaira, K., et al. (2013)	LC	IHC > 25%	anti-GLUT1(AB15309); polyclonal antibody; Abcam
Kang, S. S., et al. (2002)	BC	IHC > 0	anti-GLUT1(NR); polyclonal antibody; DAKO
Kim, B. W., et al. (2013)	CC	IHCscore ≥ 8	anti-GLUT1(SPM498); NeoMarkers
Kitamura, K., et al. (2011)	HC	IHC score > 0	anti-GLUT1(A3536); DAKO
Kunkel, M., et al. (2003)	OSCC	IHC > 50%	anti-GLUT1(MYM); polyclonal antibody; Chemicon
Kunkel, M., et al. (2007)	OSCC	IHC > 65%	anti-GLUT1(MYM); polyclonal antibody; Chemicon
Lidgren, A., et al. (2008)	RCC	NR	anti-GLUT1(NR); monoclonal antibody; Alpha Diagnostic International
Mayer, A., et al. (2005)	CC	IHC score ≥ 1	anti-GLUT1(MYM); polyclonal antibody; DakoCytomation
Mori, Y., et al. (2007)	SGC	IHC ≥ 15%	anti-GLUT1(A3536); polyclonal antibody; DAKO
Ohba, S., et al. (2010)	OSCC	IHC score ≥ 6	anti-GLUT-1(OH-217); polyclonal antibody; IBL, Co., Ltd
Osugi, J., et al. (2015)	LC	IHC > 50%	anti-GLUT1(A3536); polyclonal antibody; DAKO
Sung, J.-Y., et al. (2010)-1	AVC	IHC ≥ 5%	anti-GLUT1(NR); polyclonal antibody; DAKO
Sung, J.-Y., et al. (2010)-2	PC	IHC ≥ 5%	anti-GLUT1(NR); polyclonal antibody; DAKO
Sung, J.-Y., et al. (2010)-3	EBDC	IHC ≥ 5%	anti-GLUT1(NR); polyclonal antibody; DAKO
Sung, J.-Y., et al. (2010)-4	GBC	IHC ≥ 5%	anti-GLUT1(NR); polyclonal antibody; DAKO
Tohma, T., et al. (2005)	ESCC	IHC > 30%	anti-GLUT1(NR); polyclonal antibody; DAKO
Younes, M., et al. (2001)	TCCB	IHC > 10%	anti-GLUT1(MYM); polyclonal antibody; Chemicon

CC: Cervical Carcinoma; HNSCC: Head and Neck Squamous Cell Carcinoma; PC: Pancreatic Cancer; BC: Breast Cancer; EOC: Epithelial Ovarian Cancer; CRC: Colorectal Cancer; OSCC: Oral Squamous Cell Carcinoma; LC: Lung Cancer; HC: Hepatocellular Carcinoma; RCC: Renal Cell Carcinoma; SGC: Salivary Gland Cancer; AVC: Ampulla of Vater Carcinoma; EBDC: Extrahepatic Bile Duct Carcinoma; GBC: Gallbladder Carcinomas; ESCC: Esophageal Squamous Cell Carcinoma; TCCB: Transitional Cell Carcinoma of the Urinary Bladder; NR: Not Reported.

### Association of GLUT1 with OS

**Figure 2:**
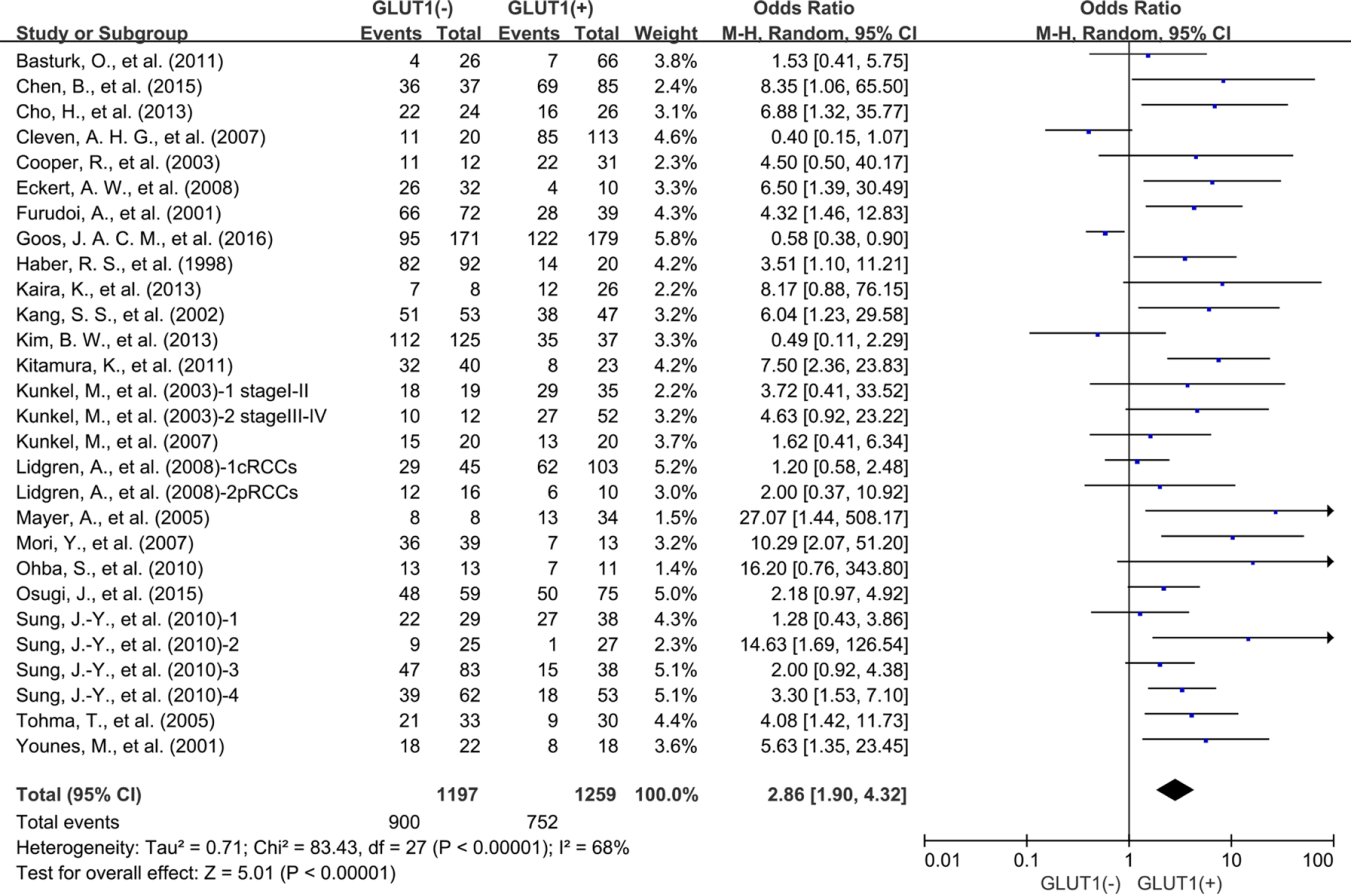
The association between expression level of GLUT1 and 3-year overall survival (OS)

**Figure 3:**
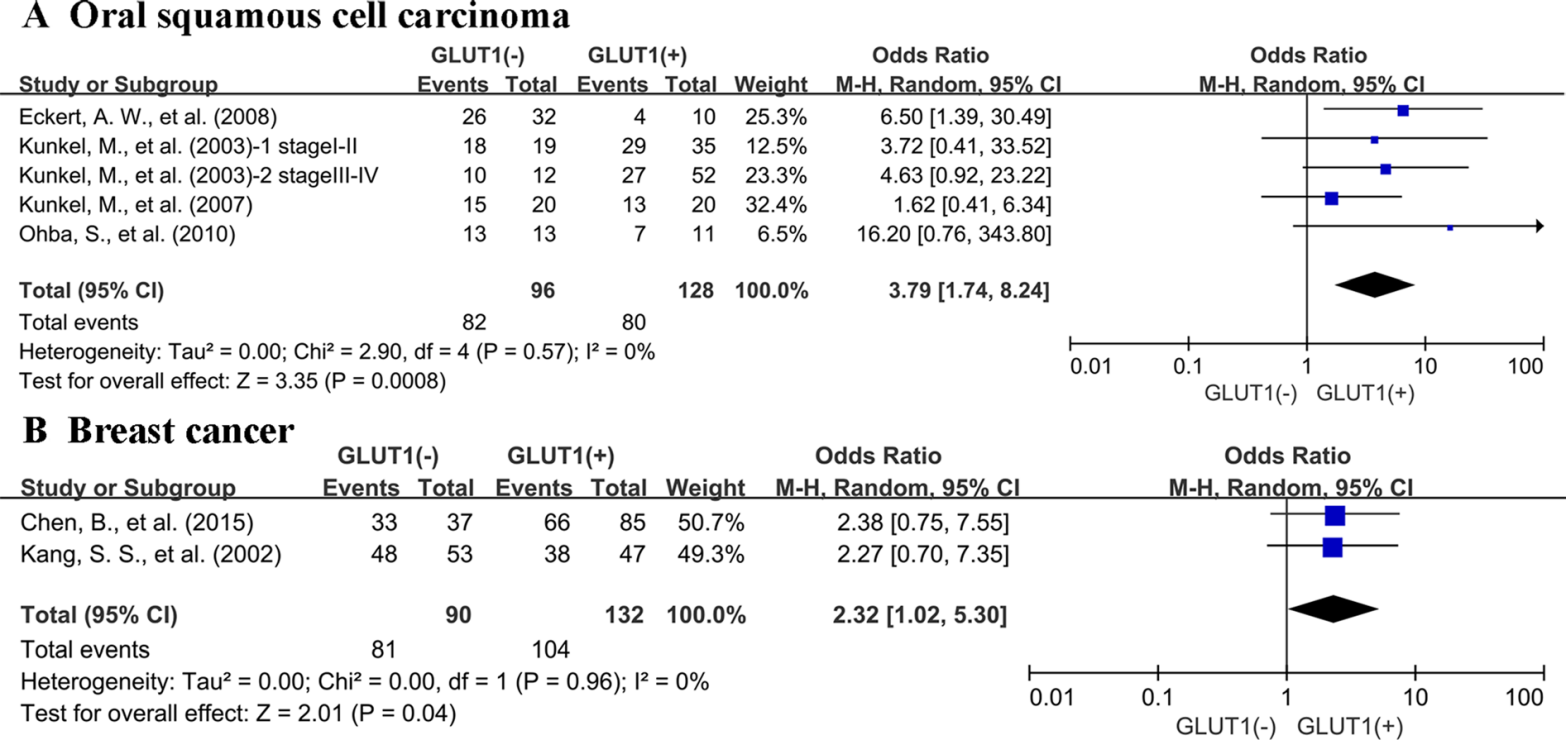
Subgroup analysis of 3-year OS by expression level of GLUT1 in different cancer types (**A**) oral squamous cell carcinoma; (**B**) breast cancer.

There were 23 studies reporting data for 3-year OS. Results revealed that GLUT1 overexpression in the tumor tissue was correlated with poor survival outcome of cancer patients (OR: 2.86; 95% CI, 1.90–4.32, *P <* 0.00001) (Figure 2). In light of high degree heterogeneity among these 23 included researches (*P <* 0.00001, I^2^ = 68%), we proceeded to perform a subgroup analysis to explore if different cancer types lead to the heterogeneity. Five researches indicated 3-year OS for colorectal carcinoma, five for oral squamous cell carcinoma, two for lung cancer, two for cervical cancer, two for breast cancer and two for pancreatic carcinoma. In the stratified analysis, expression status of GLUT1 was associated with unfavorable clinical results of oral squamous cell carcinoma (OR: 3.79; 95% CI, 1.74–8.24, *P* = 0.0008) (Figure 3A), and breast carcinoma (OR: 2.32; 95% CI, 1.02–5.30, *P* = 0.04) (Figure 3B). whereas, no association was found between high expression of GLUT1 and survival of colorectal carcinoma (OR: 1.50; 95% CI, 0.53–4.22, *P* = 0.45) (Supplementary Figure 1A), lung carcinoma (OR: 2.77; 95% CI, 1.02–7.51, *P* = 0.05) (Supplementary Figure 1B), cervical carcinoma (OR: 3.03; 95% CI, 0.05–176.64, *P* = 0.59) (Supplementary Figure 1C) and pancreatic carcinoma (OR: 4.04; 95% CI, 0.43–38.08, *P* = 0.22) (Supplementary Figure 1D).

**Figure 4:**
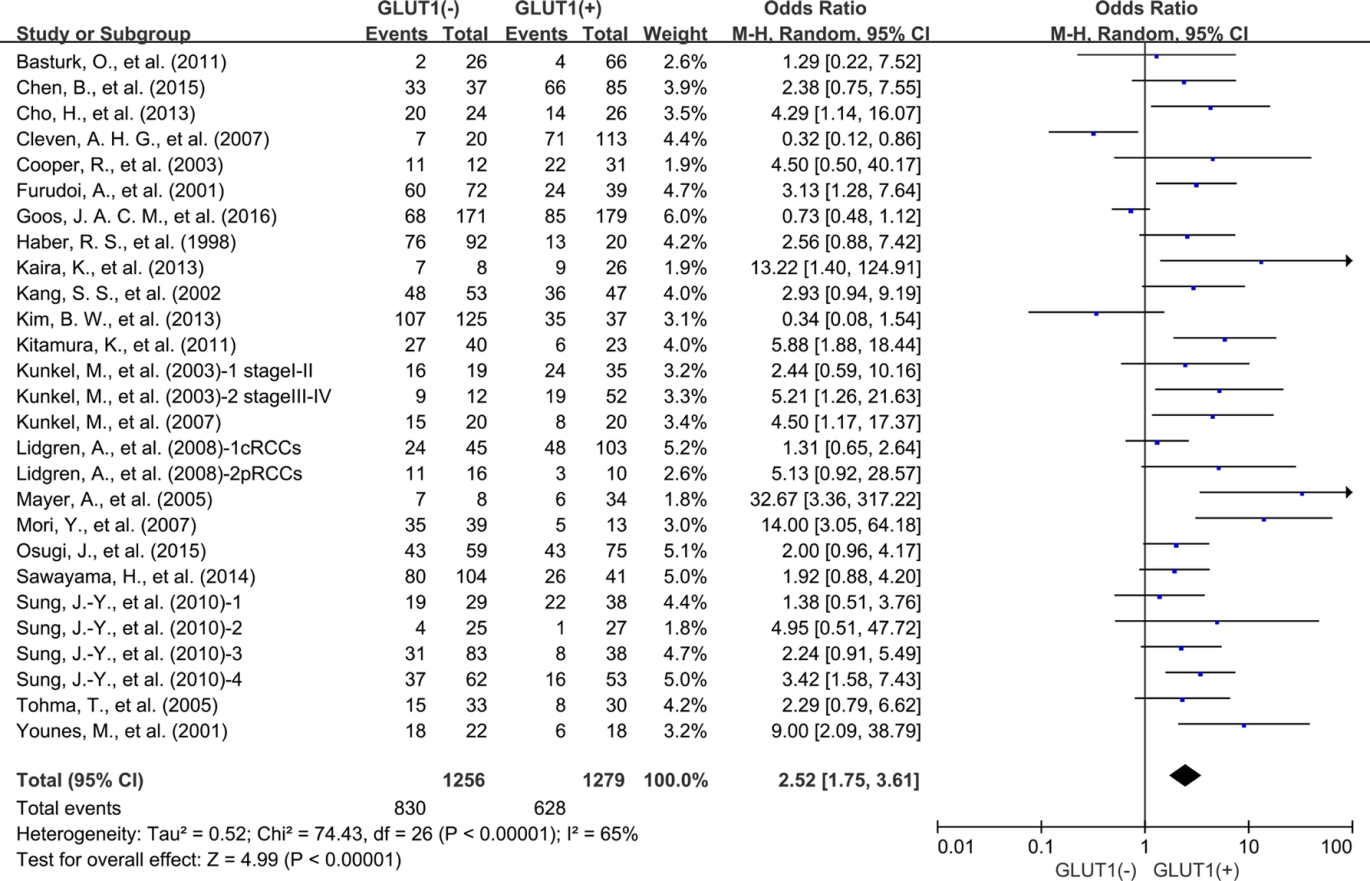
5-year OS by GLUT1 expression

**Figure 5:**
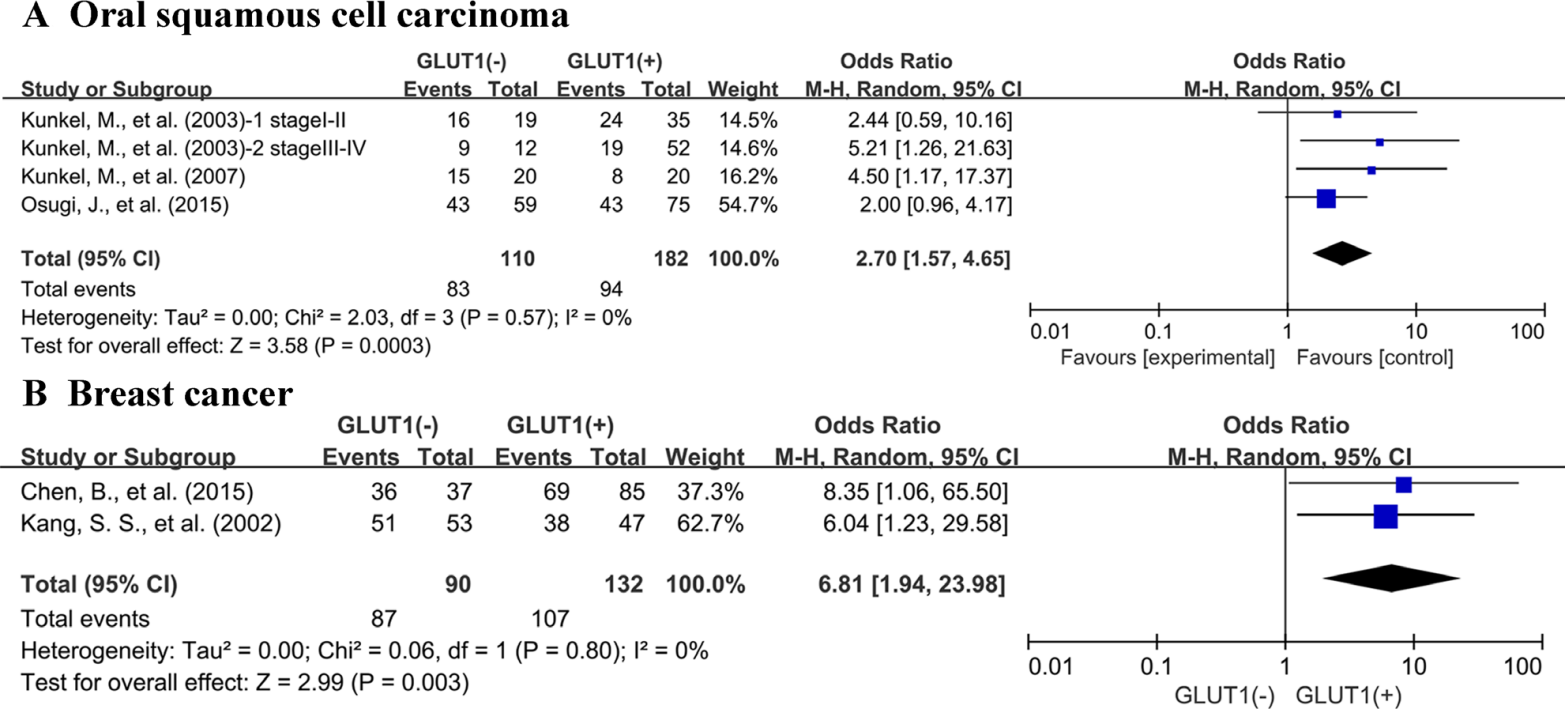
Subgroup analysis of 5-year OS by GLUT1 expression in various tumor types (**A**) oral squamous cell carcinoma; (**B**) breast cancer.

There are 21 studies presenting data for 5-years OS of cancer patients. Similar to the condition in 3-year OS, high GLUT1 expression was also correlated with unfavorable OS at 5 years (OR: 2.52; 95% CI, 1.75–3.61, *P <* 0.0000, I^2^ = 65%) (Figure 4). Because of the high degree of heterogeneity detected among these researches, we performed subgroup analysis based on various cancer types. Five researches offered 5-year OS for colorectal carcinoma, three for oral squamous cell carcinoma, two for lung cancer, two for breast cancer and two for pancreatic carcinoma. High expression status of GLUT1 was related to poor 5-year OS of oral squamous cell carcinoma (OR: 2.70; 95% CI, 1.57–4.65, *P* = 0.0003) (Figure 5A), and breast carcinoma (OR: 6.81; 95% CI, 1.94-23.98, *P* = 0.003) (Figure 5B). However, there was no correlation between overexpression level of GLUT1 and prognosis of colorectal carcinoma (OR: 1.46; 95% CI, 0.74–2.88, *P* = 0.27) (Supplementary Figure 2A), lung carcinoma (OR: 3.78; 95% CI, 0.65–22.01, *P* = 0.14) (Supplementary Figure 2B), and pancreatic carcinoma (OR: 2.14; 95% CI, 0.53–8.61, *P* = 0.28) (Supplementary Figure 2C).

**Figure 6:**
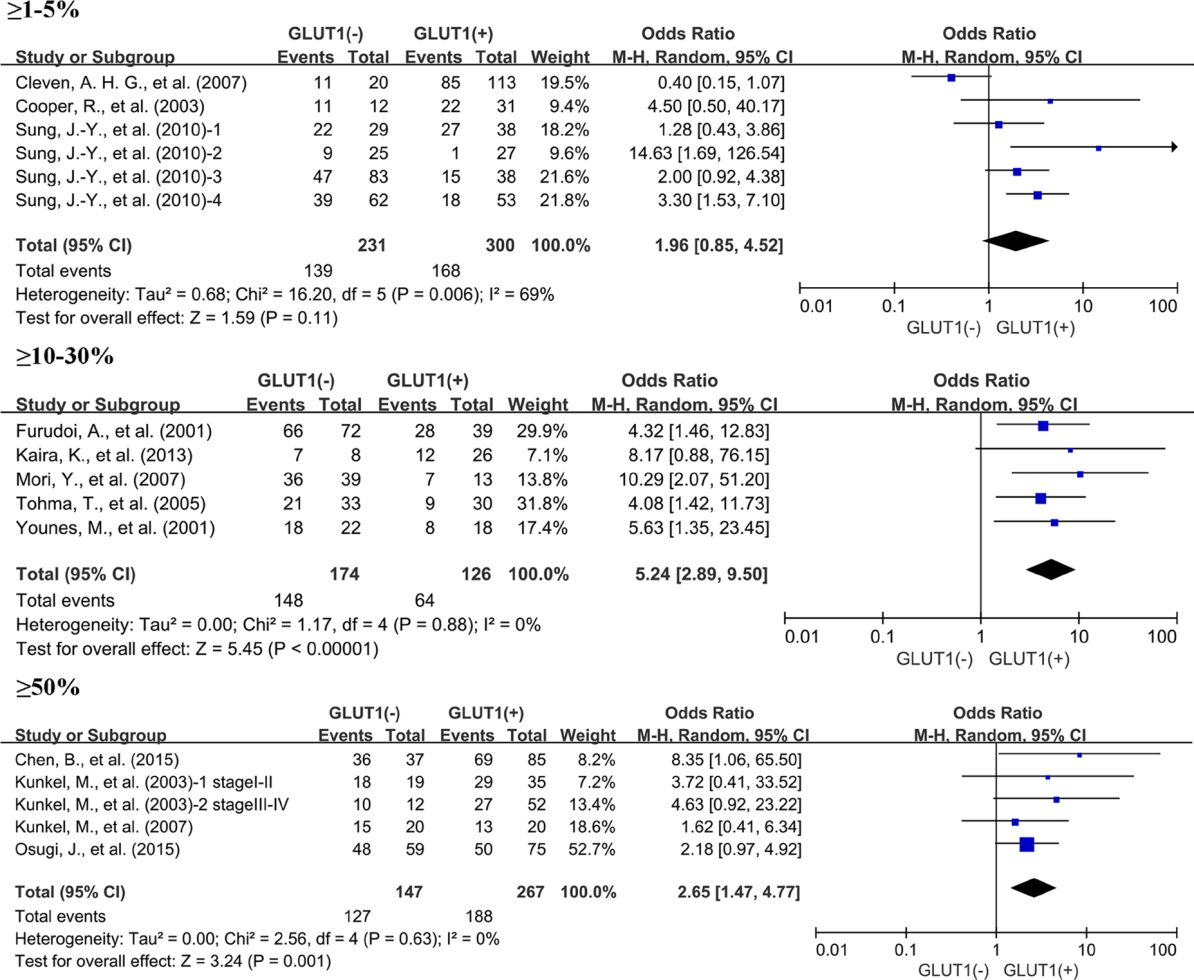
Subgroup analysis of the relationship between GLUT1 overexpression and 3-year OS of patients with solid tumors according to cut-off values identifying GLUT1 positivity

Next, we performed a subgroup analysis based on GLUT1 expression level. Results showed expression status of GLUT1 was related to unfavorable OS at 3 years in the researches using cutoff values of 10%–30% (OR: 5.24; 95% CI, 2.89–9.50, *P <* 0.00001), and 50% (OR: 2.65; 95% CI, 1.47–4.77, *P* = 0.001) (Figure 6) to determine GLUT1 positivity. Similar results were also observed in 5-year OS (Supplementary Figure 3). However, the researches used the cutoff value of 1%–5% was not correlated with 3-year OS and 5-year OS.

We have also compared the OS among studies from Asian countries and Caucasian countries based on the expression status of GLUT1. The elevated expression of GLUT1 was correlated with poor 3-year OS (OR: 3.53; 95% CI, 2.35–5.30, *P <* 0.00001) and 5-year OS (OR: 2.66; 95% CI, 1.90–3.73, *P <* 0.00001) in Asian countries. Similar results were observed when analysis of studies from Caucasian countries was performed.

In addition, analysis of four studies revealed that no correlation between GLUT1 overexpression status and 10-year OS was discovered (OR: 2.08; 95% CI, 0.83–5.20, *P* = 0.12) (Supplementary Figure 4). We also assessed the relationship between high expression of GLUT1 and the TNM stage of solid tumors. But the expression status of GLUT1 was not significantly related to TNM stage (OR: 0.72; 95% CI, 0.39–1.33, *P* = 0.30) (Supplementary Figure 5).

Meta-regression analysis showed that publication year, country, age, gender, and NOS score did not contribute to the heterogeneity (data not shown).

### Association of GLUT1 with DFS

**Figure 7:**
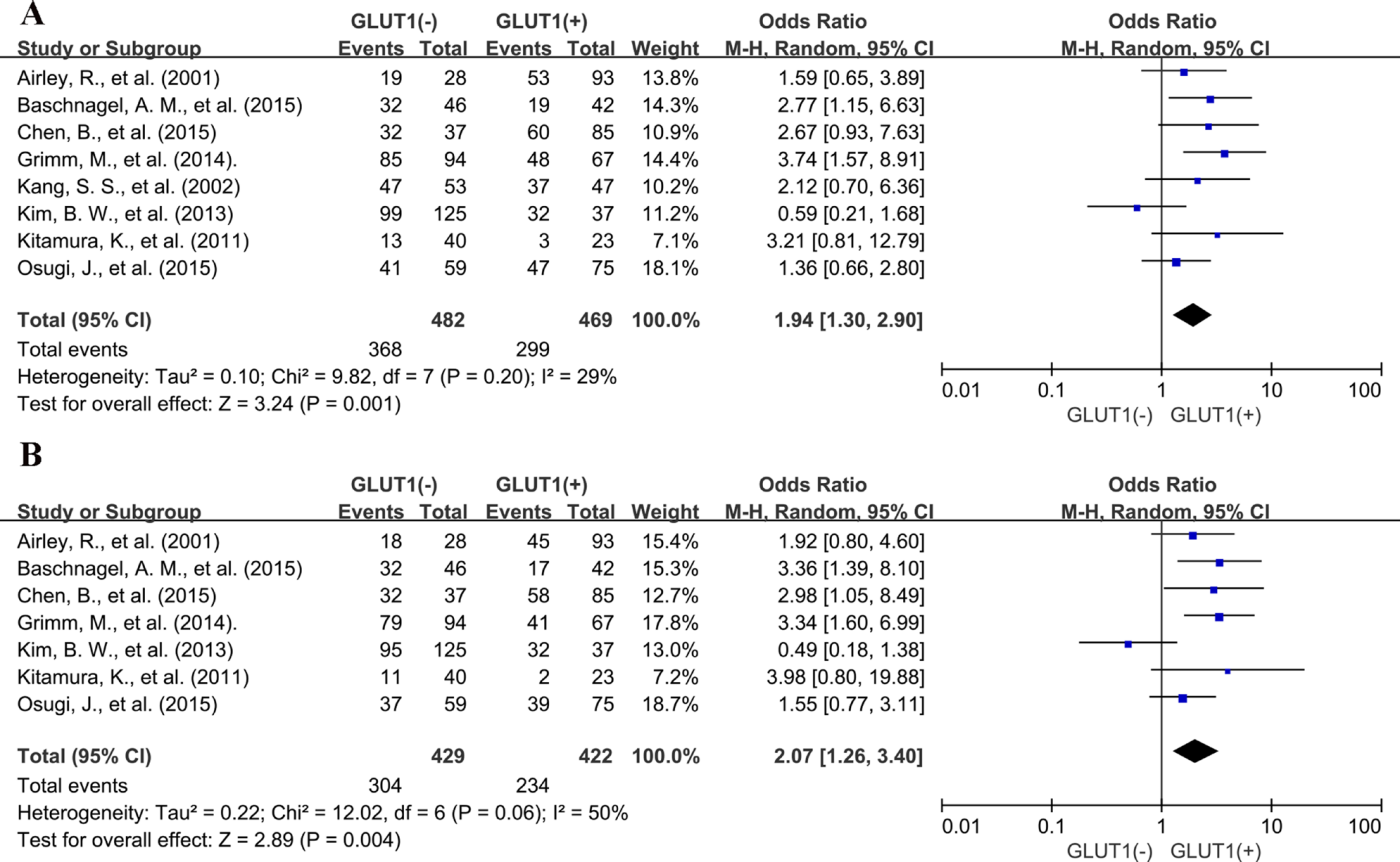
Three and five-year DFS by GLUT1 expression (**A**) 3-year DFS; (**B**) 5-year DFS.

Meta-analysis of eight studies showed that GLUT1 expression was correlated with poor 3-year DFS (OR: 1.94; 95% CI, 1.30–2.90, *P* = 0.001) (Figure 7A) and poor 5-year DFS (OR: 2.07; 95% CI, 1.26–3.40, *P* = 0.004) (Figure 7B).

**Figure 8:**
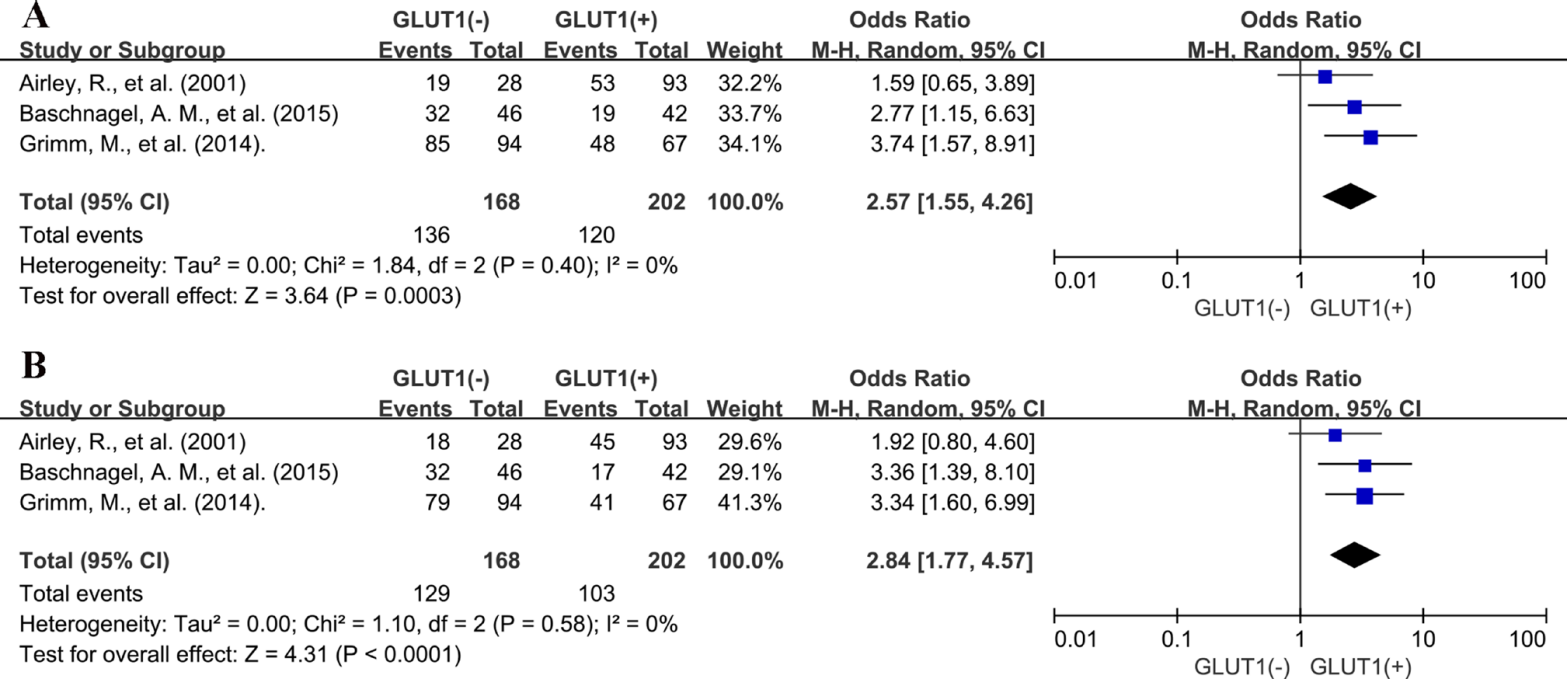
Subgroup analysis of association between GLUT1 overexpression and 3-year DFS (**A**) and 5-year DFS (**B**) in Caucasian countries.

Interestingly, no association was found between GLUT1 overexpression and 3-year, 5-year DFS (OR: 1.59; 95% CI, 0.92–2.75, *P* = 0.10; OR: 1.59; 95% CI, 0.70–3.62, *P* = 0.27) (Supplementary Figure 6) in Asian countries, whereas elevated expression of GLUT1 was significantly correlated with unfavorable DFS at 3 years (OR: 2.57; 95% CI, 1.55–4.26, *P* = 0.0003) and 5-year DFS (OR: 2.84; 95% CI, 1.77–4.57, *P <* 0.0001) in Caucasian countries. (Figure 8).

### Sensitivity analyses

Removal of the studies that was an outlier (IRS ≥ 6 or IHC > 50%) or no report (NR) with regard to the cutoff of GLUT1 overexpression by IHC did not affect outcomes for OS and heterogeneity at 3 or 5 years (OR: 3.62; 95% CI, 2.24–5.84, *P <* 0.00001, I^2^ = 54%; OR: 2.99; 95% CI, 1.94–4.60, *P <* 0.00001, I^2^ = 55%).

Exclusion of the three studies that patients did not undergo surgical treatment did not change results of OS and heterogeneity at 3 or 5 years (OR: 2.76; 95% CI, 1.83–4.15, *P <* 0.00001, I^2^ = 68%; OR: 2.38; 95% CI, 1.67–3.39, *P <* 0.00001, I^2^ = 68%, respectively). Similar results were observed when analysis of DFS was performed.

Among studies containing only surgical cases, removal of the two studies that patients received preoperative treatment and eight studies that patients underwent adjuvant therapy such as chemotherapy, radiotherapy, interferon or hormones after curative operation, did not affect consequences for OS and heterogeneity at 3 or 5 years (OR: 3.10; 95% CI, 1.82–5.28, *P <* 0.0001, I^2^ = 74%; OR: 2.54; 95% CI, 1.63–3.96, *P <* 0.0001, I^2^ = 70%, respectively). Similar results were observed when analysis of DFS was performed.

Removal of studies with NOS score 6 failed to impose impact of the results for OS at 3 years (OR: 3.30; 95% CI, 2.45–4.45, *P <* 0.00001). Omission of these studies improved heterogeneity for 3-year OS (*P* = 0.22, I^2^ = 18%). Similar results were observed in the analysis of 5-year OS.

### Publication bias

Funnel plot analysis and Begg's tests about OS and DFS at 3 and 5 years were performed to assess publication bias (Supplementary Figures 7–10). Although 5-year OS showed publication bias under Begg's test (*P* = 0.008), but no other evidences of publication bias for the studies contained in our meta-analysis were observed (3-year OS, *P* = 0.061; 3-year DFS, *P* = 1.000; 5-year DFS, *P* = 1.000). And we strictly followed inclusion criteria and criteria for protection of bias. Therefore, we considered our results is credible.

## DISCUSSION

Numerous studies have reported that GLUT1 is dysregulated in various types of human cancers [20, 23, 38], and implicated in the cancer progression and metastasis [11, 12]. But it remains unclear about the effect of GLUT1 on clinical outcomes and if the outcomes are unanimous among diverse cancer types. Our comprehensive meta-analysis of 2948 patients contained in 26 different studies indicates that the expression status of GLUT1 is a promising biomarker of unfavorable prognosis, with consistent results of OS at 3- and 5-years. Among the tumor types evaluated, overexpression of GLUT1 in tumor tissues was related with adverse OS at 3 and 5 years of oral squamous cell carcinoma and breast cancer. Our analysis found there was no significant correlation between GLUT1 overexpression and OS of colorectal cancer, lung cancer, cervical cancer and pancreatic cancer. The discordance among different types of solid tumors reveals that further researches are warranted to clarify the underlying mechanism and role of GLUT1 in pathogenesis and prognostic merit in various tumor settings.

GLUT1, one of the GLUT family, is restrictively expressed in erythrocytes and the endothelial cells of barrier tissues like blood-brain barriers, and responsible for the passive transport of glucose through the cell membrane. The full-length GLUT1 protein, with a canonical major facilitator superfamily fold, is captured in an inward-open formation [39]. Overexpression of GLUT1 is transcriptionally activated by hypoxia and hypoxia-inducible factors in glucose metabolism [40, 41]. A growing body of study has revealed that GLUT1 is dysregulated in various solid tumors, and is implicated in cancer progression and metastasis. Researches also revealed that GLUT1 expression status was correlated with 18F-FDG uptake [42], suggesting GLUT1 as a potential prognostic indicator for tumor progression or occurrence. The expression of GLUT1 in positron emission tomography (PET)-positive lesions was higher than in PET-negative ones of primary tumors as well as metastatic lymph nodes. Apart from being a potential biomarker, GLUT1 also plays a pivotal role in anticancer treatment. In light of GLUT1 as a major receptor for uptake of Vitamin C, an interesting study discovered that cultured human colorectal cancer cells with BRAF or KRAS mutations are selectively exterminated after high dose of vitamin C treatment, as a result of elevated GLUT1-facilitated uptake of the oxidized form of vitamin C, namely dehydroascorbate, which indicates a potentially novel therapy for KRAS or BRAF mutant colorectal cancers [43]. In addition, some inhibitors of GLUT1 such as fasentin [44] and histone deacetylase inhibitors [45] are potential therapeutic drugs for cancer. However, the relationship between overexpression of GLUT1 and clinical prognosis in human solid tumors remains unknown. Considering the vital role of GLUT1 both in biology mechanism and clinical application, we conducted the first meta-analysis to assess the clinical and prognostic merit of GLUT1 expression status in solid tumors.

Our meta-analysis results involve several important implications. First, it shows that GLUT1 expression is correlated to the unfavorable outcome of most solid tumors, which indicates that GLUT1 may serve as a promising therapeutic target. Second, it identifies a subgroup of tumors with adverse outcome in oral squamous cell carcinoma and breast cancer. Finally, it highlights the potential clinical application of GLUT1 as a valuable prognostic biomarker.

Several limitations also exist in our study. First, the methods and cut-off values for assessing expression status of GLUT1 are inconsistent. Second, some studies with negative results may not be published, which could cause publication bias. Lastly, substantial heterogeneity observed across eligible studies cannot be completely clarified despite appropriate meta-analytic techniques with random-effects models are used.

In this meta-analysis performed, our results show that GLUT1 overexpression in solid cancers, as evaluated by IHC, is correlated with an unfavorable prognosis in various types of tumors, suggesting that directly targeting GLUT1 could be promising therapeutic approaches for solid malignancies.

## MATERIALS AND METHODS

This meta-analysis was conducted in light of the Preferred Reporting Items for Systematic Reviews and Meta-Analyses (PRISMA) statement [46]. This study was based on the analysis and summary of the results of previously published studies, so there is no need for the ethical approval.

### Search protocol

We conducted a thorough search of Pubmed and Web of Science for studies measuring expression of GLUT1 and survival in patients with solid tumors from 1993 to April 2016. The search terms “GLUT1” and “neoplasms” were used and the results were restricted to human studies of solid tumors. A total of 944 and 934 entries were identified, respectively. Inclusion criteria were the measurement of GLUT1 by immunohistochemistry (IHC), availability of survival data for at least 3 years, and publication in English. Studies assessing gene expression of GLUT1 measured by polymerase chain reaction were excluded. We reviewed the citation lists of retrieved articles to ensure sensitivity of the search strategy. Study selection was based on the correlation of GLUT1 and clinical outcome. Inter-reviewer agreement was assessed by Cohen's kappa coefficient. Any disagreements between assessors were resolved by consulting a third assessor until a final consensus was reached.

### Endpoints of interest

Overall survival (OS) at 3, 5 and 10 years was recorded as the primary outcome of interest, and disease-free survival (DFS) at 3 and 5 years was recorded as the secondary clinical outcomes. Tumors were classified by GLUT1 expression status using cut-offs as defined by each study.

### Data extraction process and quality assessment

The following details were independently extracted by two authors (JW and CYY): name of first author, publication year, country, type of cancer, the number of patients, median age, gender, time of follow-up, cut-off value to determine GLUT1 positivity, and antibody used for the evaluation. OS data were extracted from the tables or Kaplan-Meier curves for both GLUT1 negative and GLUT1 positive group. The studies included in this meta-analysis were all cohort studies. Two authors independently measured the quality of each included study by Newcastle-Ottawa Scale (NOS) [47]. A consensus NOS score for each study was achieved by discussion. 6 scores or more were taken to denote studies of high quality.

### Data synthesis

The relative frequency of OS at 3-, 5-, 10-year and DFS at 3-, 5-year between GLUT1 negative and GLUT1 positive group was reported as an odds ratio (OR) and its 95% confidence interval (CI). Sensitivity analysis was performed for different analytical methods and NOS scores for quality evaluation of included studies.

### Statistical analysis

Data extracted from the primary publications were analysed by RevMan 5.3 analysis software (Cochrane Collaboration, Copenhagen, Denmark). Estimates of ORs were weighted and pooled using the Mantel–Haenszel random effect model. Statistical heterogeneity was evaluated with the Cochran's Q and I^2^ statistics. Differences between subgroups were assessed in accordance with the Cochrane Handbook for Systematic Reviews of Interventions [48]. Meta-regression analysis was performed by Stata 12.0 software (StataCorp LP, College Station, TX). All statistical tests were two-sided, and statistical significance was defined as *P* less than 0.05.

## SUPPLEMENTARY FIGURES


