# Supplementary material for: Glucose transporter GLUT1 expression and clinical outcome in solid tumors: a systematic review and meta-analysis
Source: Oncotarget. 2017 Feb 7;8(10):16875–86. doi: 10.18632/oncotarget.15171 (PMC5370007; doi:10.18632/oncotarget.15171)
Supplement: Supplementary file 1 [file oncotarget-08-16875-s001.pdf]

Glucose transporter GLUT1 expression and clinical outcome in solid tumors: a systematic review and meta-analysis

Supplementary Materials

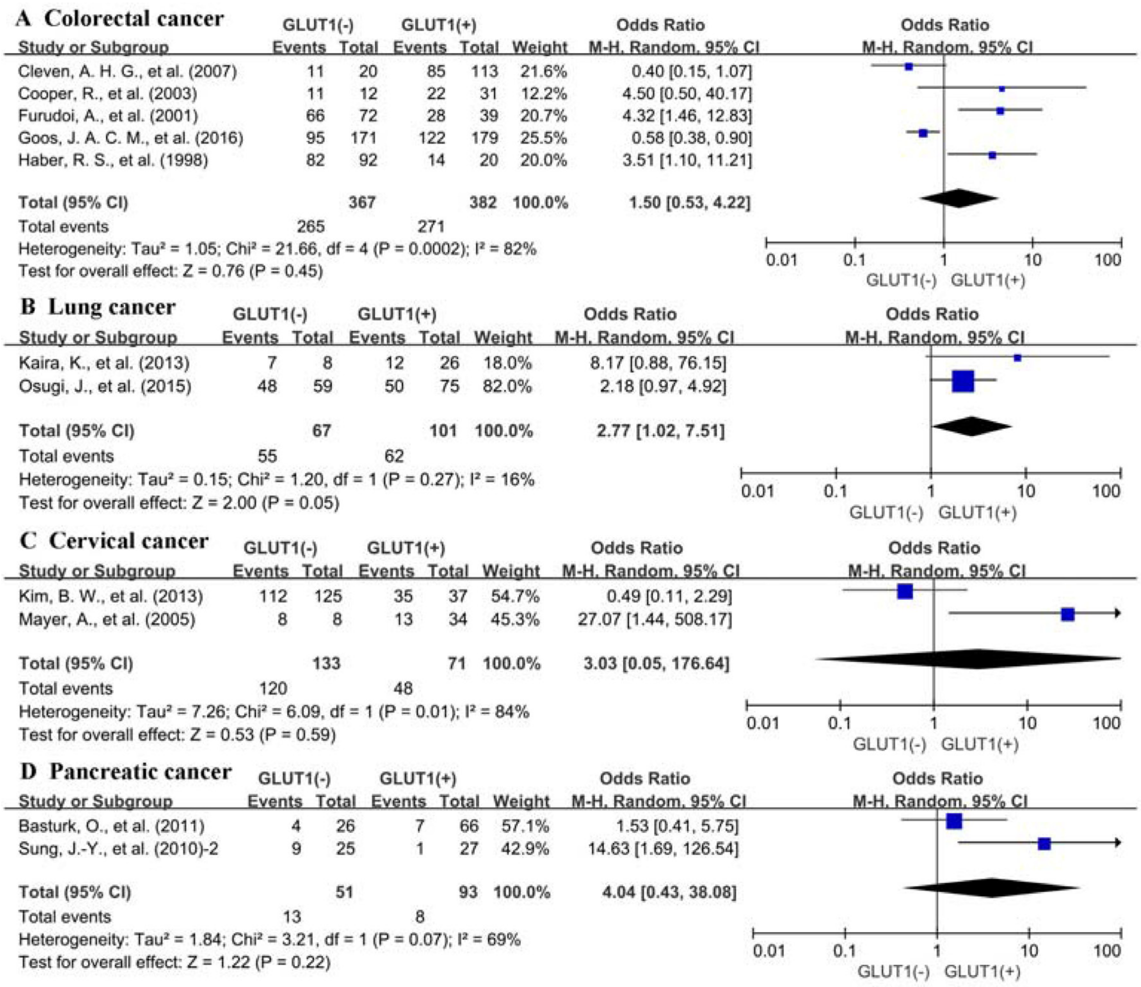

Supplementary Figure 1: Subgroup analysis of 3-year OS by GLUT1 expression in different tumor types. (**A**) colorectal cancer; (**B**) lung cancer; (**C**) cervical cancer; (**D**) pancreatic cancer.

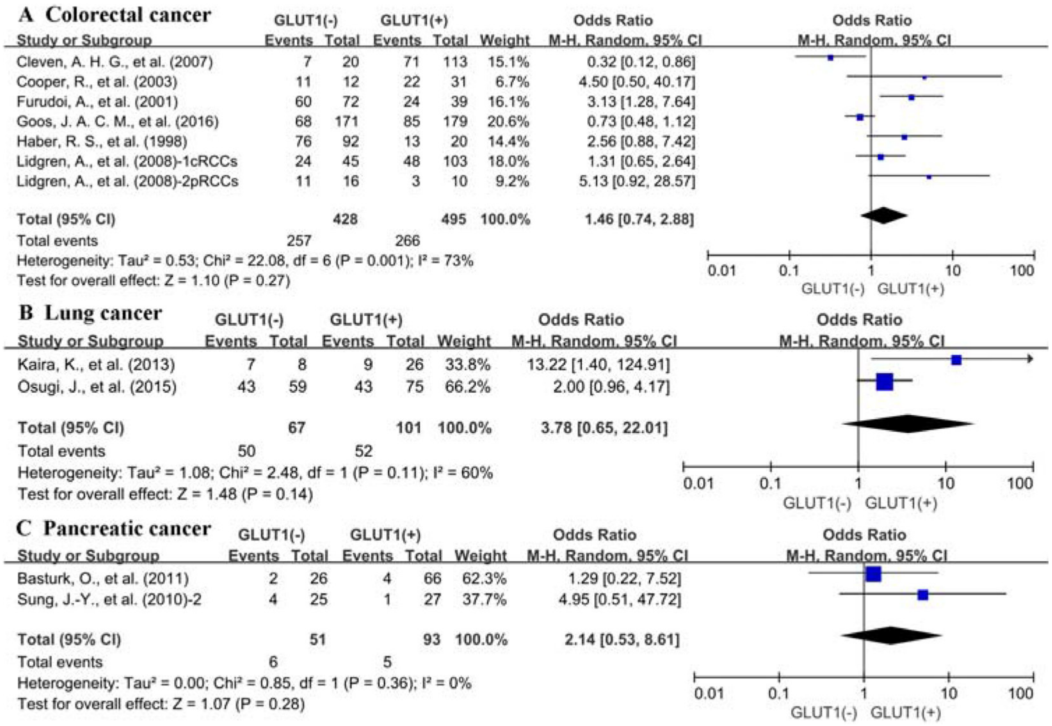

Supplementary Figure 2: Subgroup analysis of 5-year OS by GLUT1 expression in different tumor types. (**A**) colorectal cancer; (**B**) lung cancer; (**C**) pancreatic cancer.

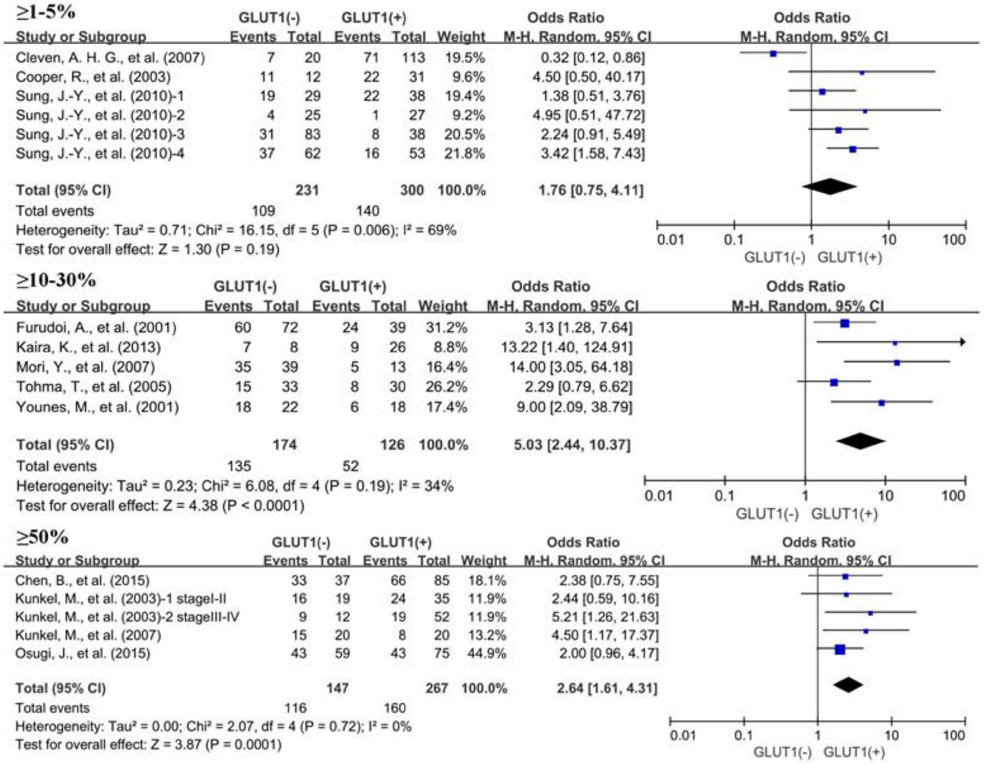

Supplementary Figure 3: Subgroup analysis of the correlation between GLUT1 overexpression and 5-year OS of solid tumors according to cut-off values determining GLUT1 positivity.

Study or Subgroup	GLUT1(-) Events	GLUT1(-) Total	GLUT1(+) Events	GLUT1(+) Total	Weight	Odds Ratio M-H, Random, 95% CI
Cleven, A. H. G., et al. (2007)	5	20	68	113	14.2%	0.22 [0.07, 0.65]
Kunkel, M., et al. (2003)-1 stageI-II	17	19	11	35	11.4%	18.55 [3.63, 94.63]
Kunkel, M., et al. (2003)-2 stageIII-IV	5	12	12	52	13.0%	2.38 [0.64, 8.88]
Lidgren, A., et al. (2008)-1cRCCs	21	45	42	103	16.0%	1.27 [0.63, 2.57]
Lidgren, A., et al. (2008)-2pRCCs	11	16	3	10	10.9%	5.13 [0.92, 28.57]
Sung, J.-Y., et al. (2010)-1	11	29	15	38	14.7%	0.94 [0.35, 2.53]
Sung, J.-Y., et al. (2010)-2	2	25	1	27	7.8%	2.26 [0.19, 26.60]
Sung, J.-Y., et al. (2010)-3	23	83	2	38	12.0%	6.90 [1.54, 31.01]
**Total (95% CI)**		**249**		**416**	**100.0%**	**2.08 [0.83, 5.20]**
Total events	95		154			

Heterogeneity: Tau² = 1.23; Chi² = 28.97, df = 7 (P = 0.0001); I² = 76%

Test for overall effect: Z = 1.57 (P = 0.12)

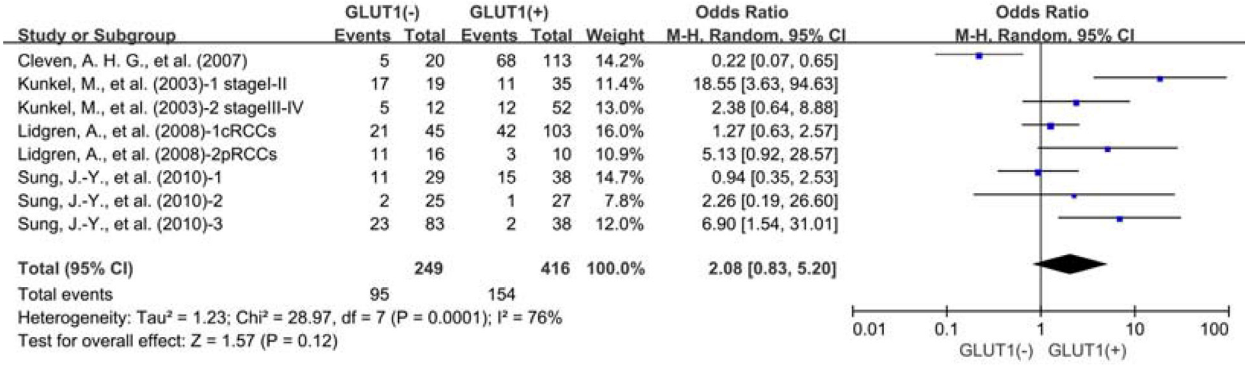

Supplementary Figure 4: 10-year OS by GLUT1 expression.

Study or Subgroup	TNM stage I-II Events	TNM stage I-II Total	TNM stage III-IV Events	TNM stage III-IV Total	Weight	Odds Ratio M-H, Random, 95% CI
Baschnagel, A. M., et al. (2015)	25	57	21	40	12.3%	0.71 [0.31, 1.59]
Basturk, O., et al. (2011)	46	63	20	29	11.3%	1.22 [0.46, 3.19]
Chen, B., et al. (2015)	31	70	11	52	12.3%	2.96 [1.31, 6.70]
Eckert, A. W., et al. (2008)	0	22	2	20	3.1%	0.16 [0.01, 3.64]
Grimm, M., et al. (2014)	21	84	46	77	13.2%	0.22 [0.11, 0.44]
Lidgren, A., et al. (2008)-1cRCCs	38	56	65	92	12.9%	0.88 [0.43, 1.80]
Lidgren, A., et al. (2008)-2pRCCs	3	15	7	11	6.8%	0.14 [0.02, 0.83]
Lidgren, A., et al. (2008)-3chRCCs	0	7	1	6	2.7%	0.24 [0.01, 7.21]
Sung, J.-Y., et al. (2010)-1	36	65	2	2	3.2%	0.25 [0.01, 5.36]
Sung, J.-Y., et al. (2010)-2	24	46	3	6	7.1%	1.09 [0.20, 5.98]
Sung, J.-Y., et al. (2010)-3	35	107	3	14	9.0%	1.78 [0.47, 6.80]
Sung, J.-Y., et al. (2010)-4	51	111	2	4	5.9%	0.85 [0.12, 6.25]
**Total (95% CI)**		**703**		**353**	**100.0%**	**0.72 [0.39, 1.33]**
Total events	310		183			

Heterogeneity: Tau² = 0.63; Chi² = 31.48, df = 11 (P = 0.0009); I² = 65%

Test for overall effect: Z = 1.04 (P = 0.30)

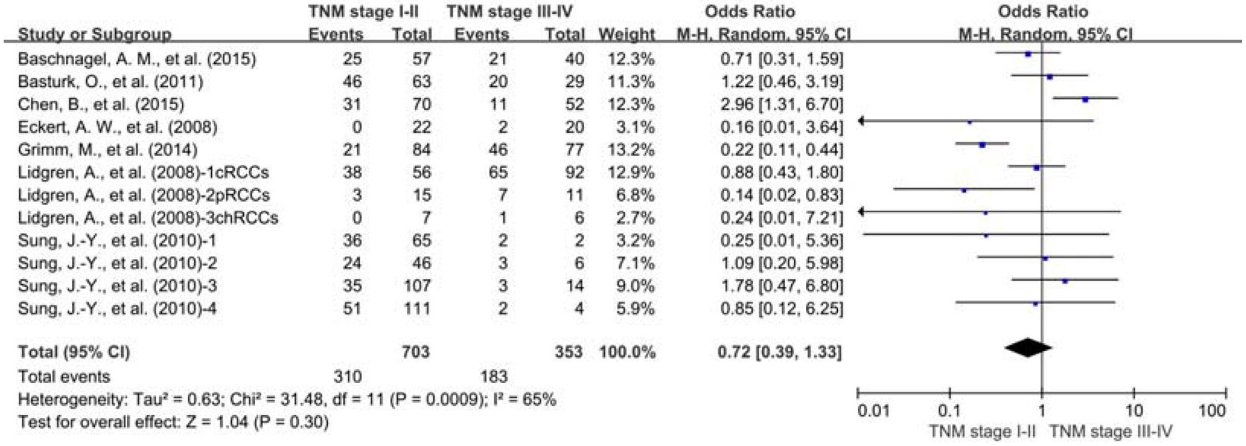

Supplementary Figure 5: Subgroup analysis of the correlation between GLUT1 expression and tumor stage.

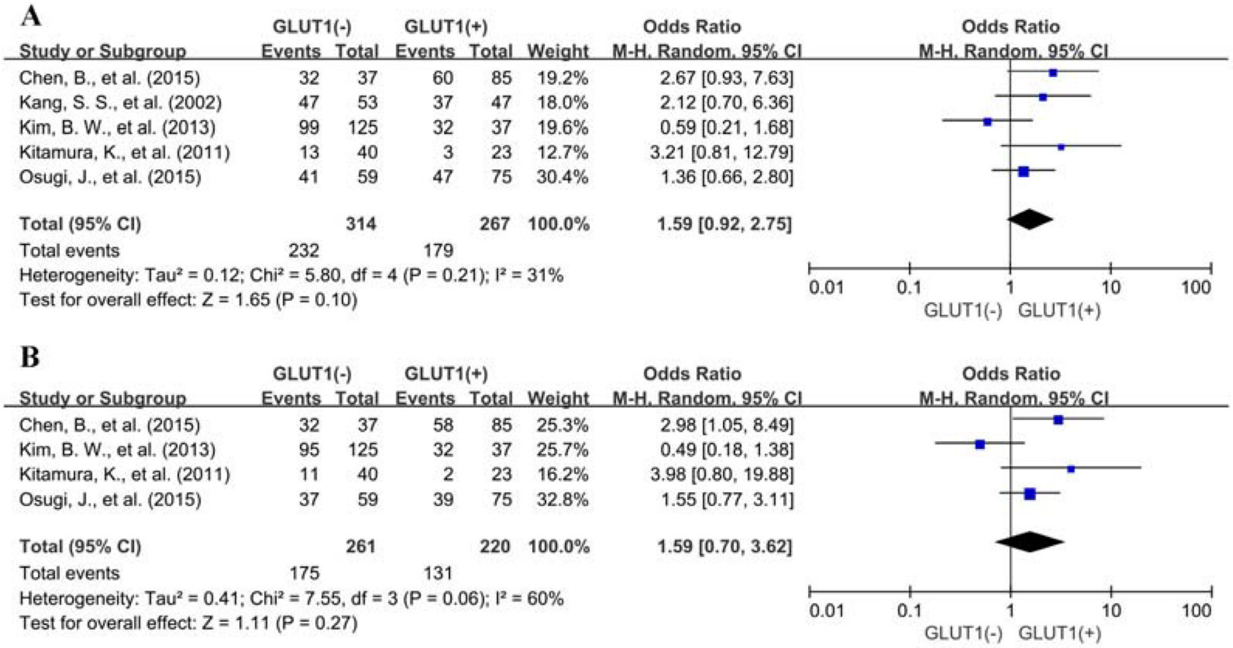

Supplementary Figure 6: Subgroup analysis of association between GLUT1 overexpression and 3-year DFS. (**A**) and 5-year DFS (**B**) in Asian countries.

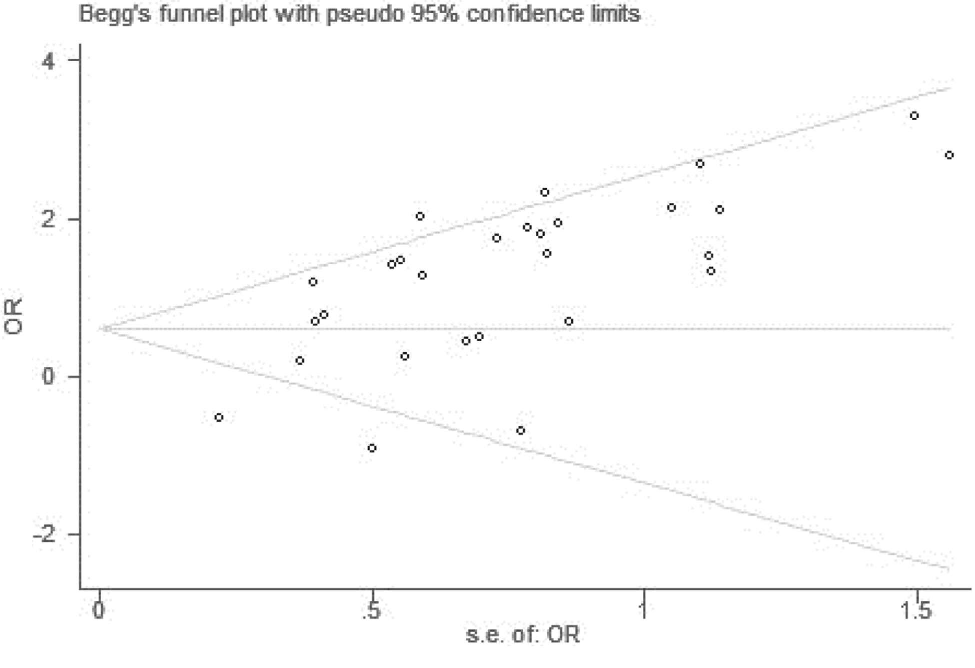

Supplementary Figure 7: Assessment of publication bias using Begg's funnel plot analysis of studies on 3-year OS.

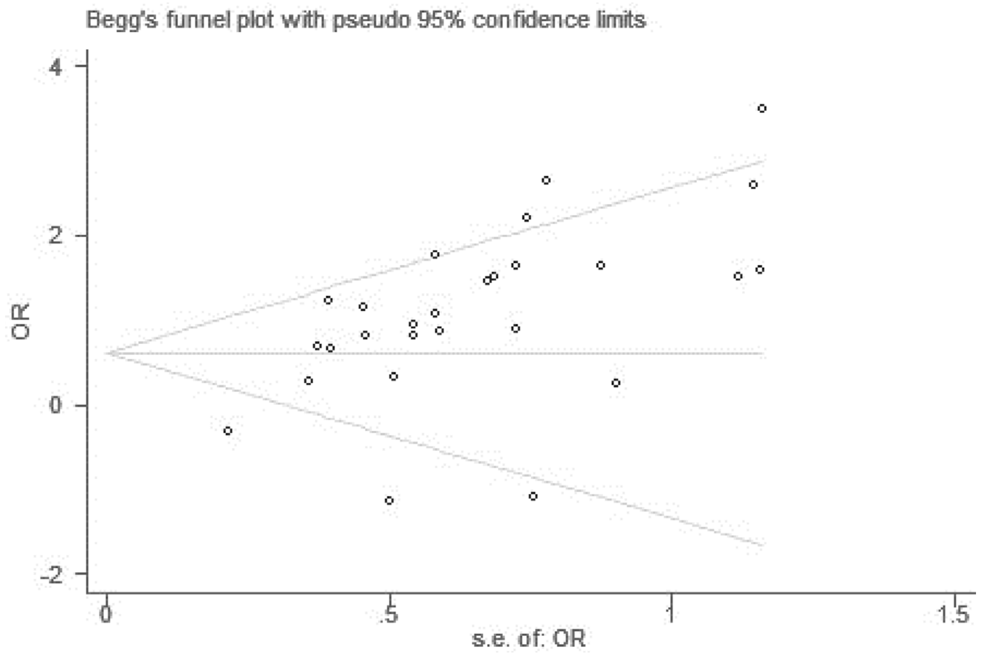

Supplementary Figure 8: Assessment of publication bias using Begg's funnel plot analysis of studies on 5-year OS.

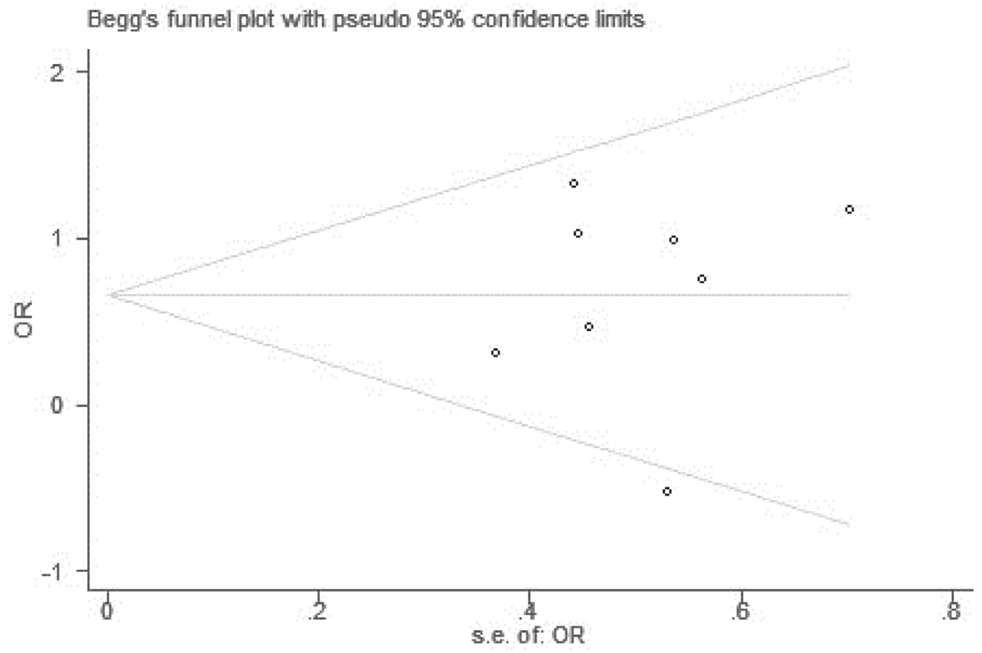

Supplementary Figure 9: Assessment of publication bias using Begg's funnel plot analysis of studies on 3-year DFS.

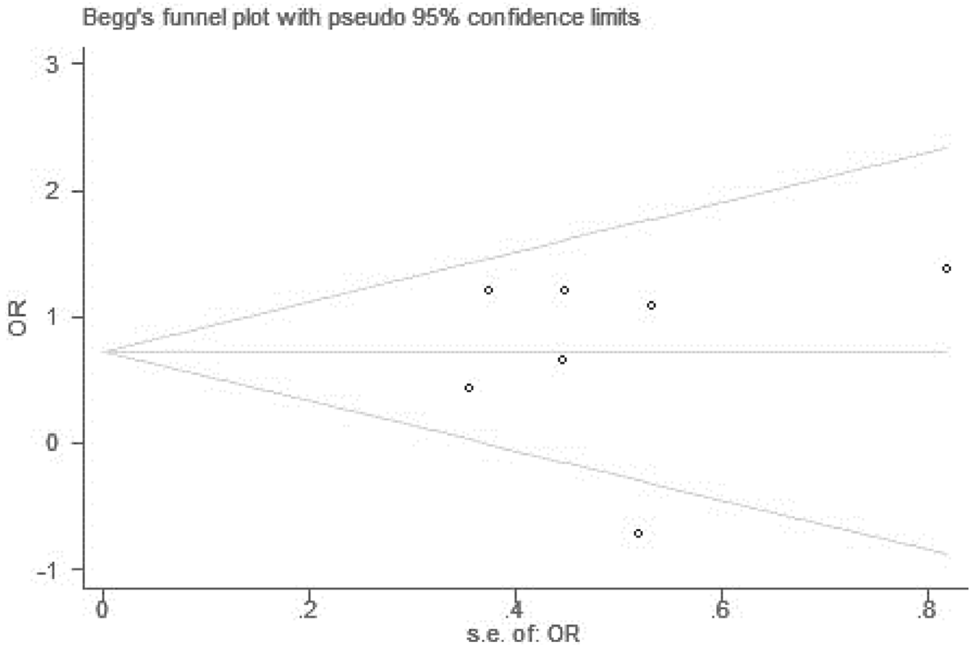

Supplementary Figure 10: Assessment of publication bias using Begg's funnel plot analysis of studies on 5-year DFS.
